# Engineering Organ-on-a-Chip Systems for Cancer Immunotherapy: Strategies and Assay Integration

**DOI:** 10.3390/bioengineering13050492

**Published:** 2026-04-23

**Authors:** Jie Wang, Zongjie Wang

**Affiliations:** 1 Chan Zuckerberg Biohub Chicago, Chicago, IL 60642, USA; jie.wang@czbiohub.org; 2 Department of Biomedical Engineering, Northwestern University, Evanston, IL 60208, USA

**Keywords:** organ-on-a-chip, immunotherapy, immune cells, immunological assays

## Abstract

Translating preclinical findings into effective clinical cancer immunotherapies remains a major challenge, mainly because conventional in vitro and animal models often fail to capture the complexity, dynamics, and species-specific features of human immune responses. Organ-on-a-chip (OoC) technologies that combine engineered tissue architectures with precisely controlled microfluidic transport provide human-relevant microphysiological platforms for mechanistic studies of immune–tumor interactions and evaluation of therapeutic efficacy and immunotoxicity under defined microenvironmental conditions. However, immune responses involve time-dependent and interconnected processes, including immune cell trafficking, cytokine programs, metabolic shifts, and cytolysis, that are not adequately resolved by static or endpoint assays. Engineering immune-competent OoC systems therefore requires coordinated design of platform architectures, immune cell incorporation strategies, and integrated measurement workflows capable of capturing dynamic and state-dependent responses. In this review, we summarize engineering strategies for building immune-competent OoC platforms for cancer immunotherapy, focusing on platform architectures, immune cell incorporation methods, and fit-for-purpose assay workflows. Emphasis is placed on embedded sensing modalities (e.g., cytokine, oxygen, and impedance readouts) that provide valuable kinetic and state-variable data. Finally, we discuss key translational challenges, including reproducibility, standardization, and benchmarking, and outline near-term priorities to accelerate the adoption of immune-competent OoC systems in immunotherapy research and development.

## 1. Introduction

Cancer remains a leading cause of global mortality, despite the continuous introduction of novel clinical therapies. Among these, cancer immunotherapy that harnesses the immune system’s ability to recognize and eliminate malignant cells has reshaped modern oncology and offered durable, and in some cases curative, clinical benefits [1,2]. Landmark clinical studies in advanced metastatic melanoma and other malignancies have demonstrated the transformative potential of immune-based therapies [3,4].

In spite of these successes, translating immunotherapy concepts into broadly effective and safe clinical products remains challenging [2,5,6]. Unlike conventional cytotoxic chemotherapies, immunotherapies rely on multistep, context-dependent processes such as immune cell trafficking, activation, cytokine signaling, and dynamic interactions within heterogeneous tissue microenvironments. Moreover, different classes of immunotherapies operate under distinct biological mechanisms. For example, immune checkpoint inhibitors restore T cell function by blocking inhibitory signaling pathways such as the PD-1/PD-L1 axis, thereby reversing T cell exhaustion. In contrast, adoptive cell therapies, including CAR-T cells, rely on ex vivo engineered immune cells that traffic to, infiltrate, and persist within tumor tissues. Other approaches, such as oncolytic viruses, bispecific and monoclonal antibodies, induce anti-tumor immunity through selective targeting and immune-mediated tumor cell killing. As a result, therapeutic outcomes depend not only on immune status, tumor architecture, and local biochemical microenvironments, but also on the specific therapeutic modality. These coupled factors make immunotherapeutic responses difficult to predict and contribute to long development timelines, high costs, and relatively low approval rates [6]. Recent estimates suggest that development of a single immunotherapy can cost up to $5 billion [7], with late-stage clinical trials (Phase II and III) representing the largest financial burden. Failures at these stages can lead to losses of hundreds of millions of dollars. These financial risks underscore the urgent demand for improved early-stage predictive models capable of evaluating efficacy and toxicity before costly clinical progression [8].

Current preclinical evaluation primarily relies on in vitro two-dimensional (2D) cell culture and in vivo animal models. While these systems have supported decades of drug development, both exhibit substantial limitations in predicting clinical outcomes for immunotherapies [9,10]. Conventional 2D cultures lack the key features of human tissues, such as three-dimensional (3D) architectures, multicellular interactions, biomechanical cues, and transport gradients characteristic of native tissues [11]. In oncology, monolayer models often fail to capture diffusion barriers and spatial heterogeneity presented in tumors, where extracellular matrix density, irregular vasculature, and metabolic gradients modulate immune infiltration and drug penetration [12]. Animal models, although capable of capturing systemic biological complexity, are constrained by high cost, low throughput, ethical considerations, and, most critically, species-specific differences in immune biology [13]. Such immunological disparities frequently limit translational relevance and contribute to discrepancies between preclinical and clinical outcomes [13,14,15].

To bridge this gap, a range of in vitro 3D tissue models have been developed to emulate human pathophysiology and reduce reliance on animal studies [16]. Models such as spheroids and scaffold-based cultures introduce improved architectural complexity and cell–cell interactions. However, most remain static systems governed primarily by diffusion-driven transport. As construct thickness increases (typically ~1–2 mm), passive diffusion becomes inadequate to sustain oxygen and nutrient delivery, resulting in hypoxic or necrotic regions that compromise long-term tissue viability and experimental reproducibility [17,18]. These limitations suggest the need for dynamic platforms that integrate controlled perfusion and microenvironmental regulation.

Organ-on-a-chip (OoC) technologies provide such dynamic capabilities through integration of engineered 3D tissue constructs with microfluidic control. Continuous perfusion, defined extracellular matrices, and mechanical stimulation enable these systems to recapitulate key biochemical and biophysical features of human tissue microenvironments over extended culture durations [19]. A broad range of organ-specific platforms, including lung-on-a-chip and vascularized tumor-on-a-chip systems, have been developed to evaluate drug efficacy, toxicity, and underlying mechanisms under physiologically relevant conditions [20]. Coupling multiple organ modules through vascular perfusion or supernatant exchange additionally allows modeling of organ–organ interactions and systemic pharmacological effects, therefore offering a systems-level perspective on disease progression and therapeutic response [8,21].

With these technological advances, regulatory agencies have increasingly promoted “new approach methodologies” (NAMs), including microphysiological systems such as OoC platforms, to complement or reduce reliance on animal studies [22]. Despite growing interest in human-relevant test systems, OoC applications in cancer immunotherapy remain relatively underdeveloped. Immune responses are inherently dynamic, multiscale, and sensitive to spatial organization, transport conditions, and measurement strategies. Incorporating immune components into microfluidic tissue systems therefore presents unique engineering challenges beyond conventional OoC design.

**Figure 1:**
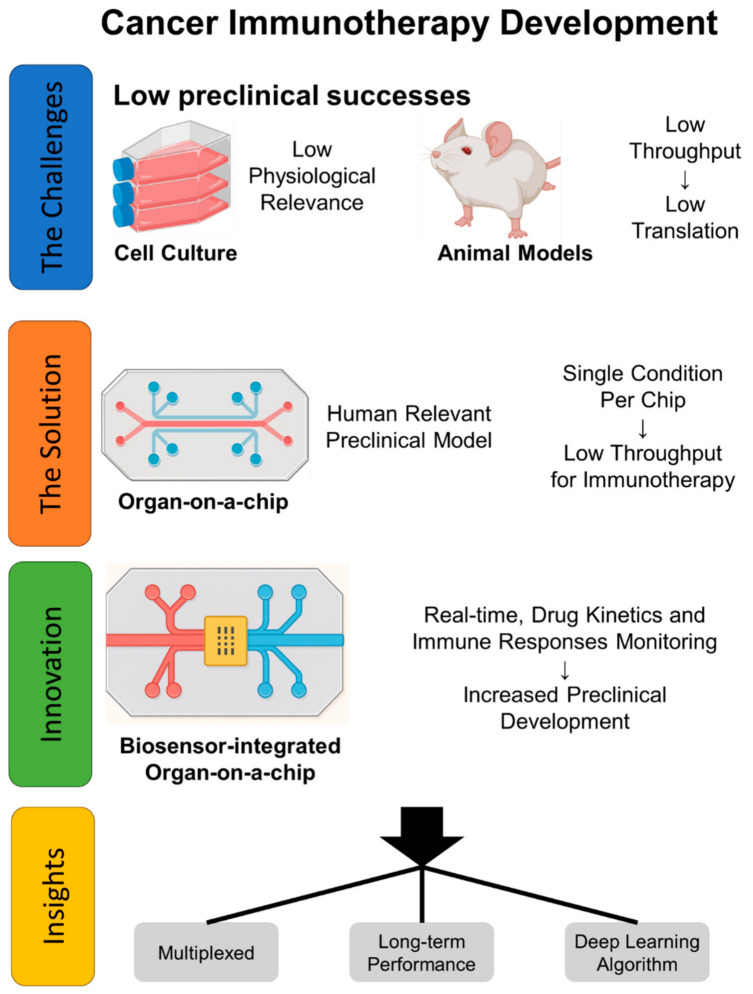
Conceptual overview of challenges and engineering opportunities in cancer immunotherapy modeling. Challenges: Conventional 2D cultures and animal models often fail to capture key aspects of human physiology and immune–tumor dynamics. Approach: Organ-on-a-chip (OoC) platforms integrate microfluidics and tissue engineering to recreate human-relevant microenvironments with controlled transport and multicellular organization. Assay integration: Coupling OoC with quantitative readouts—including imaging, effluent assays, and selected integrated sensors—enables time-resolved interrogation of immune signaling, microenvironmental state, and functional outcomes. Outlook: Progress in multiplexed measurements, long-term device operation, and data integration/analysis workflows is advancing the translational utility of OoC platforms for immunotherapy development.

In this review, we focus on engineering principles and workflows for developing immune-competent OoC systems for cancer immunotherapy research. Here, immune-competent refers to an OoC platform that incorporates functional immune components capable of recapitulating key processes such as immune cell trafficking, activation, and effector responses within a physiologically relevant tissue microenvironment. We discuss strategies for immune–tissue integration, microenvironment control, and quantitative functional readouts. We further summarize immunological assays used in OoC and outline practical considerations for standardization, robustness, and fit-for-purpose validation in immunotherapy development (Figure 1). While OoC designs have been reviewed extensively elsewhere [23,24,25], our objective is to synthesize immune-specific engineering principles and highlight recent advances most relevant to immunotherapy applications.

## 2. Engineering Evolution of Immune-Competent OoC Platforms

**Figure 2:**
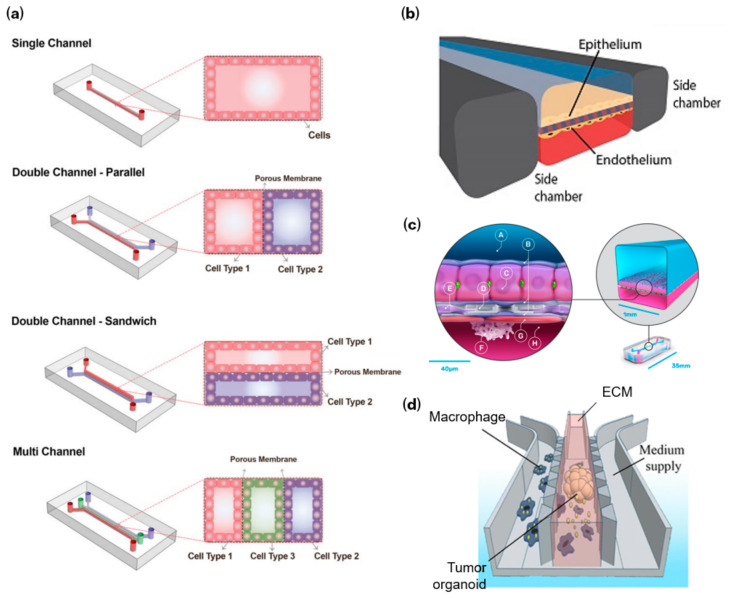
Representative microfluidic architectures for OoC platforms. (**a**) Common OoC microfluidic layouts, including single-channel systems, dual-channel “parallel” configurations separated by a porous membrane, dual-channel “sandwich” configurations that mimic layered tissue interfaces, and multi-channel platforms with three or more interconnected compartments to model more complex microenvironments. Reproduced with permission from Tajeddin et al. [27]. (**b**) Canonical two-channel lung-on-a-chip/gut-on-a-chip architecture illustrating an epithelial–endothelial interface separated by a flexible porous membrane, with lateral vacuum chambers enabling cyclic stretch. Reproduced with permission from Huh et al. [28]. (**c**) Liver-on-a-chip model supporting immune and inflammatory interactions. Primary human hepatocytes (C) are embedded in extracellular matrix (B) on a porous membrane (D) in the upper parenchymal channel (A), while liver sinusoidal endothelial cells (G), Kupffer cells (F), and stellate cells (E) are cultured on the opposite side within the lower vascular channel (H). This configuration enables studies of inflammatory signaling and immune–tissue interactions in a perfused microenvironment. Reproduced with permission from Ewart et al. [29]. (**d**) Organoid-on-a-chip configuration in which a 3D tumor organoid embedded in extracellular matrix (ECM) is perfused by medium and interfaces with immune cells (e.g., macrophages), illustrating integration of 3D tissue constructs with multicellular interactions. Reproduced with permission from Stavrou et al. [30].

The development of OoC technologies for immunotherapy has progressed through distinct engineering phases, marked by increasing physiological realism and functional integration. Early systems focused on microfluidic control of flow and gradients; subsequent platforms introduced organ-level barrier function under perfusion and mechanical cues; more recent designs incorporate immune components with quantitative and translationally oriented readouts [26]. Representative single-, dual- (parallel and sandwich), and tri-channel architectures are summarized in Figure 2a.

### 2.1. Foundational Microfluidics Enabling Immune-Relevant Transport (2000 to 2014)

Early microfluidic systems focused on precise control over flow profiles, chemical gradients, and shear stress, enabling reproducible studies of immune cell migration and stimulus–response behavior under well-defined boundary conditions [31,32]. This progress laid the transport “operating layer” for later immune–tissue co-culture. The subsequent emergence of organ-mimetic chips, such as epithelial and endothelial barrier models maintained under dynamic flow and mechanical strain, extended microfluidic engineering into tissue-level function [33,34] (Figure 2b). By coupling barrier integrity with controlled immune exposure, these systems provide a framework for studying leukocyte adhesion, transmigration, and cytokine-driven inflammation within spatially organized geometries. Although immune integration at this stage remained limited, the essential architectural principles for immune–barrier interaction studies were established.

### 2.2. Organ-Level Biomimicry with Immune Integration and Quantitative Readouts (2014 to 2021)

The next generation of OoC platforms underscored improved physiological fidelity. Integration of primary and iPSC-derived cells, ECM-supported 3D architectures, and sustained perfusion enabled longer-term and more biologically representative tissue cultures [35]. Importantly, this period marked a shift toward routine quantitative measurements. Techniques such as TEER [36] and related barrier-function readouts, helped transition immune studies from descriptive imaging toward quantitative evaluation of inflammatory signaling, immune–endothelial interactions, and tumor–immune crosstalk [37,38] (Figure 2c). Multi-module and recirculating systems gained prominence during this period [39,40], which supported controlled immune trafficking between compartments and distributed responses across interconnected tissues [41,42]. While still primarily mechanistic, these systems began to approximate pharmacokinetic and pharmacodynamic hypotheses within a human-relevant microenvironment.

### 2.3. Translation-Oriented Platforms: Standardization, Immune Modules, and NAM Context (2022 to Present)

More recently, focus has shifted toward translational readiness and operational robustness. Engineering priorities now include standardized workflows, reproducible operating modes, and modular immune components that are compatible with pharmacology-style designs. In parallel, regulatory and funding agencies have underlined “new approach methodologies” (NAMs), including OoC platforms, as part of this transition [43]. Commercial systems, such as Emulate, TissUse, and Mimetas, now increasingly demonstrate the ability to incorporate circulating immune cells and inflammatory contexts, which reflects growing demand for immunotoxicity and cytokine-related assessment workflows [44]. Looking forward, the field is expected to prioritize reproducibility, multiplexed quantitative endpoints (not necessarily continuous biosensing), and integration of patient-derived tissues such as organoids and hydrogel-supported explants to support more predictive, fit-for-purpose immunotherapy evaluation (Figure 2d; Table 1).

## 3. Immune Cell Incorporation Strategies for Physiological Immunotherapy Modeling

To make OoC systems immunotherapy-relevant, immune cells must be integrated in ways that preserve immune function, spatiotemporal signaling, and physiologic transport between tumor, stromal, and vascular compartments. A critical biological constraint in the integration is human leukocyte antigen (HLA) compatibility. Antigen-specific T cell responses highly depend on HLA matching, and the use of allogeneic immune cells may result in non-specific activation or failure to recognize tumor-associated antigens. Autologous systems, in which tumor cells and immune cells are derived from the same donor, provide greater physiological relevance but introduce additional complexity in sample acquisition and experimental standardization. These constraints suggest that immunological compatibility is not merely a biological consideration but a central design factor that influences how immune cells can be introduced, maintained, and functionally engaged within OoC platforms. In practice, immune integration can be framed as an engineering problem defined by three coupled variables: (i) delivery mode (bolus addition vs. continuous perfusion/recirculation), (ii) compartment geometry (co-mixed vs. segregated), and (iii) barrier design (open interface, porous membrane, epithelial barriers, or endothelialized vasculature). These variables collectively determine exposure kinetics, cytokine gradients, and the balance between sustained immune engagement and premature exhaustion. Spatial separation of tumor, stromal, and immune compartments can mitigate premature immune exhaustion while preserving structural cues necessary for realistic immune–tumor interactions [52,53]. Micro-engineered barriers with controlled perfusion further enable selective communication through cytokine diffusion, chemotactic gradients, and regulated cellular migration [39]. In current immunotherapy-oriented OoC designs, immune cells are typically introduced following ex vivo preparation workflows, including peripheral blood mononuclear cell isolation, optional cryopreservation and recovery, and T cell activation/expansion [54,55]. These cells are then incorporated via vascular perfusion channels or compartmentalized microfluidic architectures separated by porous or endothelial barriers. Each configuration captures distinct aspects of tumor immunobiology.

### 3.1. Vascular Perfusion Models

**Figure 3:**
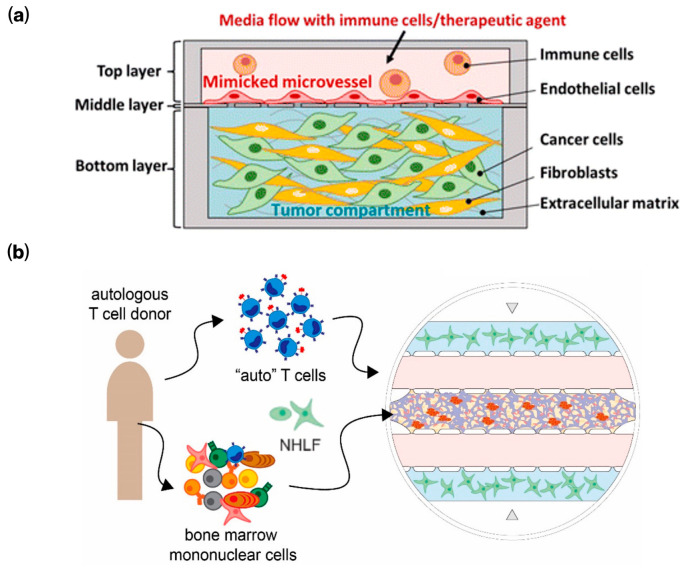
Immune cell incorporation strategies for immunotherapy-oriented microphysiological models. (**a**) Vascular perfusion model: Immune effector cells are introduced through an endothelialized perfusion channel under controlled flow, enabling adhesion, transmigration across the vascular barrier, and infiltration into a tumor compartment containing cancer cells, fibroblasts, and ECM to emulate immune trafficking. Reproduced with permission from Chi et al. [58]. (**b**) Compartmentalized microfluidic architecture: Vascularized multi-compartment bone-marrow-on-a-chip with distinct endothelialized vascular and stromal (normal human lung fibroblast) niches separated by a porous interface. CAR-T cells are perfused through the vascular channel and subsequently transmigrate into the 3D marrow compartment to mediate antigen-specific killing. Reproduced with permission from Ghoshal et al. [60].

Vascular perfusion models aim to replicate immune trafficking and infiltration by incorporating endothelialized channels under physiologically relevant flow conditions. In these systems, circulating effector cells (e.g., T cells, NK cells) are introduced into perfused vascular channels, where they adhere to activated endothelium, transmigrate across barrier layers, and infiltrate adjacent tumor or stromal compartments [56,57]. This configuration captures critical early steps of immunotherapy response, including adhesion dynamics, extravasation efficiency, and flow-dependent immune engagement. Because perfusion continuously replenishes immune cells and removes secreted factors, these models are particularly well suited for studying trafficking kinetics and vascular barrier interactions. For example, Chi et al. engineered a recirculating tumor-on-a-chip platform that sustained continuous T-cell circulation and quantitatively visualized migration across an endothelial barrier into the tumor microenvironment under flow (Figure 3a) [58]. More recently, Liu et al. developed a perfused transplantation platform for supporting patient-derived tumor explants with CAR T cells in vitro [59]. By preserving native tumor architecture and vasculature, the system enabled analysis of heterogeneous CAR T penetration and context-dependent expression of cytotoxicity and exhaustion markers. These findings demonstrate the importance of microenvironmental and transport constraints on therapeutic efficacy. From an engineering perspective, vascular perfusion models are advantageous for outlet sampling and longitudinal cytokine analysis but require careful tuning of shear stress and flow rate to avoid non-physiologic immune activation or washout.

### 3.2. Compartmentalized Microfluidic Architectures

Compartmentalized architectures spatially segregate tumor and immune regions using porous membranes or epithelial/endothelial barriers. These configurations preserve compartment-specific microenvironments while permitting controlled paracrine signaling and chemotaxis through regulated diffusion and directed migration [61]. They also enable longer-term interrogation of immune polarization (e.g., macrophage M1/M2 switching) and checkpoint dynamics while maintaining stable tissue organization [62]. For example, Ghoshal et al. developed a microvascularized human bone marrow model comprising distinct endosteal and perivascular compartments, through which donor-matched CAR-T cells were perfused via a vascular channel into the marrow compartment (Figure 3b) [60]. This configuration enabled simultaneous assessment of CAR-T survival, differentiation, infiltration, cytokine secretion, and cytotoxic efficacy within a controlled niche. Compartmentalized chips preserve tissue organization over extended culture durations. However, they introduce increased fabrication complexity and require careful design to avoid artificial gradient artifacts.

### 3.3. Summary

Immune cell incorporation strategies define the spatial organization, exposure kinetics, and functional lifespan of immune responses in OoC systems. Vascular perfusion models enable dynamic trafficking and continuous exposure, whereas compartmentalized architectures preserve localized microenvironmental signaling and longer-term immune–tumor interactions. However, these configurations inherently constrain accessible readouts, sampling strategies, and temporal resolution. As a result, immune incorporation cannot be decoupled from downstream measurement design. This interdependence motivates a unified framework in which platform architecture and assay selection are co-optimized, as discussed in later sections.

## 4. Classic Immunological Readout Assays for OoC

Established immunological assays, including microscopy-based imaging, soluble-factor quantification, and single-cell phenotyping, have been adapted to OoC platforms for routine measurement of immune responses in vitro. In immune-competent OoC, however, readout selection is constrained not only by analytical goals but also by chip architecture, sampling access, assay invasiveness, and the biological time scale under investigation (e.g., rapid killing kinetics versus multi-day phenotype evolution). In this section, we summarize commonly used readouts in OoC immunotherapy studies and highlight practical considerations for their implementation.

### 4.1. Fluorescent Imaging

Fluorescent imaging relies on fluorophores, molecules that absorb light at one wavelength (excitation) and emit light at a longer wavelength (emission). When illuminated with the appropriate excitation light, fluorophores produce signals that can be captured by detectors, enabling visualization of cell morphology, spatial organization, and biomarker expression relevant to disease progression and therapeutic response [41,63,64,65]. In immune-competent OoC, fluorescence imaging is particularly valuable because it preserves spatial context. By labeling tumor and immune populations with fluorophores, researchers can visualize immune cell trafficking, infiltration depth, cell–cell contact, and tumor killing dynamics within tissue-like microenvironments. Live/Dead imaging and viability assays are frequently used to assess the direct effects of immunotherapy on cancer cell survival through quantification of cell death and overall treatment response.

However, imaging in OoC platforms faces significant technical limitations, including photobleaching, optical distortion, and restricted penetration depth, issues that are particularly pronounced in thick 3D matrices [66]. Most importantly, image-derived data are complex and often require sophisticated analysis pipelines to translate qualitative observations into reproducible quantitative metrics [66]. Many fluorescence-based protocols require staining steps that can be disruptive to cells and tissues, particularly during fixation and permeabilization, which can potentially compromise native structures (e.g., DNA) and limit downstream functional studies.

### 4.2. ELISA

Beyond imaging-based readouts, biochemical and immunological assays provide complementary function information by measuring secreted biomarkers associated with immune activation, suppression, and inflammation. Among these, ELISA and related cell-based assays enable detection of cytokines and signaling molecules released by tissues and immune cells, thereby supporting mechanistic interpretation of on-chip immune responses to immunotherapies [35,41,67]. ELISA is based on specific antigen–antibody interactions, in which target molecules are captured by immobilized antibodies and detected by enzyme-linked secondary antibodies to generate a measurable signal. Because ELISA typically measures secreted factors in culture media, it can be implemented without physically disrupting cells or tissues within the device. Consequently, cytokine profiling can serve as a minimally invasive and sensitive approach for identifying early immune activation or suppression and for evaluating pathway modulation in response to immunotherapy.

Nevertheless, conventional ELISA typically quantifies a single analyte per assay, thereby limiting multiplexed biomarker profiling within a single experiment [68]. The procedure can also be time-consuming and labor-intensive, which constrains throughput when many conditions or time points must be evaluated. Furthermore, minimizing false-positive and false-negative results depends critically on antibody specificity and quality, although well-validated antibody pairs can be costly and are not always readily available for certain targets [69].

### 4.3. Flow Cytometry

Flow cytometry analyzes individual cells as they flow in a fluid stream through a focused laser beam. As each cell passes through the beam, it scatters light and emits fluorescence if labeled with fluorophore-conjugated antibodies, enabling simultaneous measurement of multiple parameters such as cell size, granularity, and marker expression. This technique provides high-throughput, single-cell analysis and supports identification, characterization, and sorting of cell populations based on marker expression patterns [70]. In OoC immunotherapy studies, flow cytometry is valuable for profiling immune cell states (e.g., activation, differentiation) and for quantifying heterogeneous immune subpopulations that may drive therapeutic response or resistance [70].

Despite these advantages, flow cytometry typically requires preparation of single-cell suspensions, which often necessitates removing cells from the microfluidic device and dissociating tissues or matrices. Such processing can perturb cellular phenotypes, bias recovery of specific populations, and introduce cell damage or loss, especially when extracting cells from confined microfluidic geometries or hydrogel-embedded compartments [71]. Consequently, flow cytometry is often constrained to endpoint analyses or experimental designs where cells can be recovered from perfusion channels with minimal disruption.

### 4.4. Summary

Classic immunological assays provide robust and well-validated tools for characterizing immune responses in OoC systems, but their implementation is constrained by tradeoffs in invasiveness, temporal resolution, and compatibility with microfluidic architectures, as summarized in Table 2. Live-cell imaging preserves spatial context but is analytically complex; effluent assays are accessible but temporally discrete; and flow cytometry enables deep phenotyping at the cost of disruption. These limitations suggest that assay selection is not purely analytical but fundamentally connected to platform design and operation. This constraint motivates the integration of complementary and more scalable measurement strategies, particularly those capable of capturing dynamic immune responses under continuous perfusion.

## 5. Emerging Immunological Readout Assays for OoC

To complement single-point readout assays, emerging sensing modalities have been developed and integrated into OoC platforms to enable continuous or repeated monitoring of biochemical and biophysical parameters under perfusion. Unlike single-point measurements, these approaches provide kinetic and state-variable information that is critical for interpreting immune–tumor interactions in dynamically perfused systems [83,84]. In this section, we highlight representative sensing strategies focused on cytokine secretion, oxygen dynamics, and impedance-based cytotoxicity, as shown in Table 2. These modalities illustrate how integrated sensing can improve mechanistic interpretability in immune-competent OoC experiments by linking immune activation, microenvironmental regulation, and functional killing in real time.

### 5.1. Cytokine Sensing for On-Chip Immune Activation Kinetics

Cytokines regulate immune cell proliferation, migration, and activation and serve as central mediators of cell–cell signaling in cancer and inflammation [85]. In cancer immunotherapy, cytokine programs reflect immune activation states and influence both therapeutic efficacy and inflammatory toxicity [86]. For example, effector cytokines can enhance T cell and NK cell cytotoxicity, thereby promoting anti-tumor immunity [86]. Cytokines are also used therapeutically (e.g., interleukins and interferons) to modulate immune activity, either to stimulate anti-tumor responses or to mitigate excessive inflammation [87]. Accordingly, measuring cytokine dynamics in OoC models provides mechanistic context for distinguishing productive anti-tumor activation from dysregulated inflammatory signaling.

**Figure 4:**
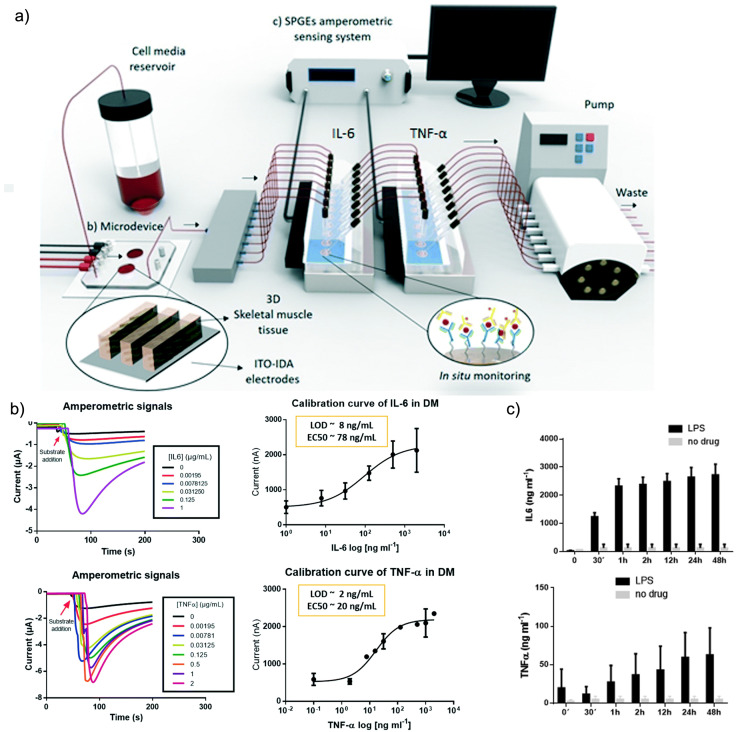
Muscle-on-a-chip with integrated electrochemical cytokine sensing. (**a**) Schematic of microfluidic workflow. Tissue constructs are stimulated electrically using indium tin oxide interdigitated array (ITO-IDA) electrodes or biologically using lipopolysaccharide (LPS). Perfusate containing secreted cytokines (IL-6 and TNF-α) is routed via an 8-way microfluidic distributor to a multiplexed sensing module. A peristaltic pump generates negative pressure to drive flow through the tissue chamber and detection system. (**b**) Representative amperometric responses and calibration curves for IL-6 and TNF-α under flow in differentiation medium. (**c**) In situ detection of IL-6 and TNF-α following LPS stimulation (10 μg mL^−1^, 48 h) in 3D skeletal muscle cultures. Reproduced with permission from Ortega et al. [72].

In most OoC studies, cytokines secreted into culture medium are quantified from collected effluents using ELISA at discrete time points. While generally accessible, this approach yields static snapshots and may fail to resolve transient secretion bursts or rapid pathway transitions during immune–tumor interactions. The limitation is particularly pronounced under continuous perfusion, where secreted factors are diluted and temporally averaged before off-chip analysis. To address this constraint, electrochemical immunosensors have been incorporated into microfluidic platforms for in situ monitoring. For example, a PDMS-based muscle-on-a-chip system was developed for localized detection of inflammatory cytokines, including IL-6 and TNF-α, secreted by murine skeletal myoblasts [72]. The device incorporated a multiplexed electrochemical sensing module at the chip outlet, using antibody-functionalized amperometric sensors with ng/mL sensitivity (Figure 4a) [72]. During operation, cytokines secreted from the tissue construct were transported by perfused medium through the microfluidic network to a screen-printed gold electrode (SPGE) amperometric sensor array, where binding to IL-6 and TNF-α antibodies generated concentration-dependent amperometric signals (Figure 4b) [72]. Using lipopolysaccharide and electrical stimulation paradigms, the platform detected stimulation-induced increases in cytokine secretion within relevant experimental windows and supported monitoring for up to approximately 48 h (Figure 4c) [72]. Building on this concept, Kanioura et al. implemented interdigitated gold electrodes functionalized with capture antibodies directly within a co-culture chamber, enabling label-free monitoring of IL-6 and IL-8 fluctuations inside the tissue microenvironment rather than exclusively at the outlet [73]. These studies demonstrated the integration of electrochemical immunosensors and OoC workflows in improving temporal-resolution cytokine readouts and reducing reliance on spatially averaged effluent sampling [72,73,74].

Despite high sensitivity and rapid response, antibody-capture cytokine sensors face practical constraints for extended monitoring. Strong antigen–antibody binding can lead to surface saturation over time, while biofouling and nonspecific adsorption may introduce signal drift under extended perfusion. In addition, outlet-only sensing configurations underrepresent spatial heterogeneity and transient gradients within 3D tissue constructs, as they primarily report bulk-averaged effluent concentrations rather than local microenvironmental dynamics. To address these limitations, emerging approaches, including integrating multiplexed electrochemical sensors into tissue scaffolds or culture chambers, aim to enhance spatial resolution and gradient mapping [88]. These developments suggest that cytokine sensing is most effective when sensor placement and affinity design are aligned with anticipated secretion flux and perfusion conditions.

### 5.2. Oxygen Sensing for Microenvironmental State Dynamics

Whereas cytokine sensing reports immune signaling outputs, oxygen monitoring captures a key microenvironmental state variable that strongly modulates both tumor biology and immune function. Within the tumor microenvironment, inadequate oxygen supply activates hypoxia-inducible pathways that drive angiogenesis, metabolic reprogramming, and therapy resistance, while tumor cells adapt by shifting toward glycolysis (Warburg effect), increasing survival and invasiveness [89]. Hypoxia also impairs anti-tumor immunity by reducing immune cell proliferation, cytotoxicity, and antigen presentation, while simultaneously promoting the accumulation of immunosuppressive populations such as regulatory T cells and myeloid-derived suppressor cells [90]. In addition, hypoxia-induced PD-L1 expression can weaken immune recognition, contributing to resistance against immune checkpoint inhibitors and reducing overall treatment efficacy [90]. Precise control and continuous monitoring of oxygen in immune-competent OoC models therefore can help determine whether observed changes in immune activation or cytotoxicity arise from therapeutic mechanisms or from microenvironmental constraints [91].

**Figure 5:**
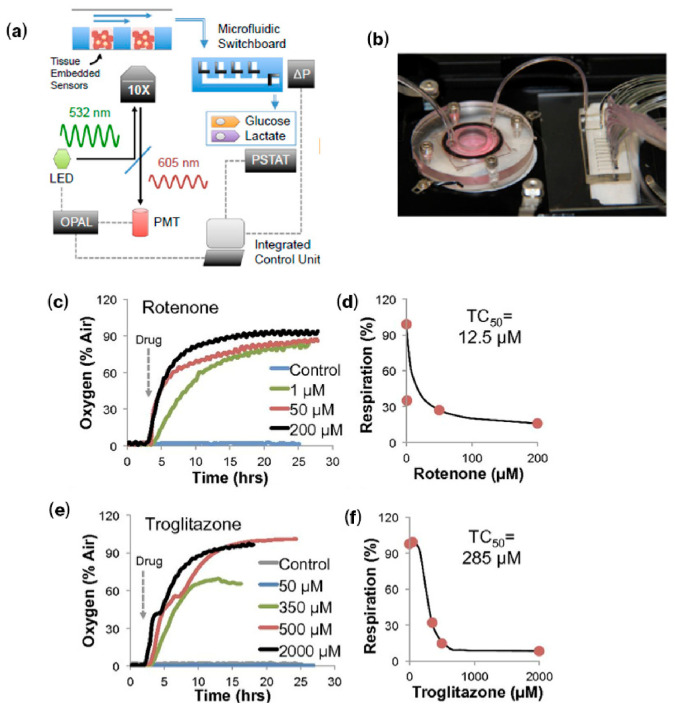
Integrated metabolic monitoring in a liver-on-a-chip bioreactor. (**a**) Sensor configuration for 3D liver microtissues: oxygen and pH are measured optically using nanoparticle-based probes embedded in the microtissues, while glucose and lactate are quantified using downstream amperometric biosensors connected via a microfluidic switchboard. (**b**) Bioreactor and switchboard setup. (**c**,**e**) Oxygen uptake traces of HepG2/C3A cells treated with increasing concentrations of rotenone (**c**) and troglitazone (**e**). (**d**,**f**) Dose-dependent effects of rotenone after 12 h (**d**) and troglitazone after 24 h (**f**); TC_50_ values were 12.5 µM (rotenone) and 285 µM (troglitazone). Reproduced with permission from Bavli et al. [75].

Oxygen levels in cell cultures are typically assessed using oxygen-sensitive dyes or ELISA-based assays at discrete time points [92]. While informative, these methods provide static snapshots and often fail to capture spatiotemporal heterogeneity under perfusion or within 3D constructs, which limits quantitative assessment of hypoxia-driven immune modulation in OoC models [92]. To address this, Bavli et al. employed an optical oxygen biosensor based on 50 µm polystyrene microbeads loaded with a ruthenium-based phosphorescent dye. The system measures oxygen-dependent phosphorescence decay using two-frequency phase modulation, a technique that ensures a robust readout independent of signal intensity, focus distance, or local probe density shifts caused by cell death and migration [75]. This system was integrated into a liver-on-a-chip platform using a polymethyl methacrylate (PMMA) bioreactor with PDMS microwell inserts (Figure 5a,b) [75]. Under physiological perfusion (2 µL/min) mimicking liver zonation, HepG2/C3A spheroids were maintained for 28 days while mitochondrial respiration was tracked in real time [75]. Exposure to rotenone (1, 50, and 200 µM) and troglitazone (50–2000 µM) caused dose-dependent declines in oxygen uptake, providing a time-resolved indicator of cellular injury (Figure 5c,f) [75].

More recently, Bouquerel et al. designed an integrated optical sensor for tumor-on-a-chip platforms by embedding a luminescent ruthenium dye within an oxygen-permeable polymer matrix positioned along the microchannel wall [76]. The sensing mechanism is based on dynamic luminescence quenching: dissolved molecular oxygen diffuses into the polymer layer and dynamically quenches the dye’s emission, resulting in oxygen-dependent reductions in luminescence intensity and lifetime [76]. By coupling the optical readout to a high-speed photodetector, the system achieved millisecond-scale temporal resolution and sub-millimeter spatial mapping of oxygen gradients across microchannels. This design permitted continuous, non-invasive monitoring of oxygen consumption by tumor spheroids under flow, with stability compatible with long-term perfusion studies [76].

Despite these advances, optical oxygen sensing requires careful integration with the broader imaging and assay framework. Excitation and emission spectra must be selected to minimize spectral overlap with fluorescent reporters, genetically encoded sensors, or autofluorescent materials within the device. For example, excitation near 532 nm and emission detection around 605 nm may interfere with commonly used fluorophores or introduce background signal from PDMS or extracellular matrices. Optical path length, scattering in 3D constructs, and phototoxicity under repeated illumination must also be considered in experimental design. Nevertheless, integrating oxygen sensing in immune-competent OoC platforms substantially enhances interpretability by coupling immune functional readouts with microenvironment state monitoring. Hypoxia is known to modulate T-cell metabolism, proliferation, and effector function and has been implicated in impaired CAR-T expansion, differentiation, and cytokine secretion in solid tumor contexts [77]. In addition, oxygen-responsive strategies have been explored to bias immune activity toward hypoxic tumor regions and potentially reduce off-tumor effects. In this context, oxygen functions not only as a monitored state variable but also as a tunable design input for immunotherapy-relevant OoC studies [78].

### 5.3. Impedance Sensing for Real-Time Label-Free Cytotoxicity Readouts

Cytotoxicity is commonly assessed using endpoint assays such as LDH, MTT, or resazurin-based assays, which quantify membrane integrity or metabolic activity at a single time point. While these methods provide quantitative measures of cell death and viability, they do not readily resolve the timing, progression, or kinetics of immune-mediated killing. In contrast, real-time impedance-based cytolytic assays enable continuous, label-free monitoring of cell coverage, morphology, and adhesion by measuring electrical impedance across embedded microelectrodes. By quantifying changes in electrical resistance at the cell–electrode interface, impedance sensors have been widely used to characterize biological structures and functions, particularly for evaluating cell layer integrity for toxicity assessment [80] and virus detection [93].

**Figure 6:**
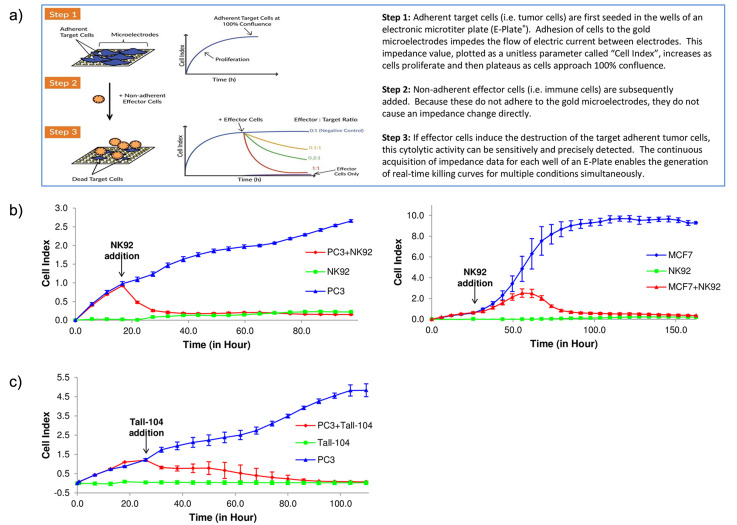
Impedance-based real-time cytolysis assay for immunotherapy potency assessment. (**a**) Step-by-step schematic illustrating impedance readout during immune-mediated killing. (**b**) Representative impedance (Cell Index, CI) traces for NK-92 effector cells against nuclear red-labeled PC3 prostate cancer cells (left) and MCF7 breast cancer cells (right). Target cells alone adhere and proliferate, increasing CI (blue), while NK-92 alone produces minimal CI change (green). Co-culture induces cytolysis and a progressive CI decline (red). (**c**) CI traces for PC3 target cells treated with the cytotoxic T cell line TALL-104, illustrating applicability to different effector cell types. Reproduced with permission from Cerignoli et al. [81].

In cancer immunotherapy studies, impedance sensors have been used to continuously monitor viability of target tumor cells under different treatment conditions [81]. A common configuration uses microtiter plates embedded with gold microelectrodes, enabling time-resolved tracking of adherent tumor cell number, morphology, and attachment strength in co-cultures that may include effector immune cells, antibodies, and small molecules [81]. Using this platform, Cerignoli et al. demonstrated a real-time immunotherapeutic potency assay with adherent tumor target lines and NK-92 effector cells. The resulting killing curves correlated closely with endpoint measurements from image-based assays and flow cytometry (Figure 6a) [81]. Mechanistically, attachment of tumor cells to the electrode surface produces a baseline impedance signal that reflects cell coverage and morphology, whereas immune-mediated cytolysis decreases impedance as target cells detach or lose barrier-like integrity (Figure 6b,c) [81]. This method therefore provides a kinetic, label-free, and non-invasive assessment of immune cell-mediated cytotoxicity without the need for fluorescent or radioactive labeling [81]. More recently, Abd Talib et al. applied an impedance-based growth monitoring approach to analyze NK-92 cytotoxicity against nasopharyngeal carcinoma models, which continuously tracks impedance across both 2D cultures and ex vivo tumor samples to infer killing dynamics in a more physiologically relevant context than single time-point assays [82].

A key limitation of impedance cytolysis assays is their reliance on physical attachment of target cells to the electrode surface. As a result, immune-mediated killing of non-adherent targets and killing events occurring in suspension or deep within 3D matrices (e.g., spheroids, organoids, or circulating tumor models), may be poorly captured because the impedance signal is primarily determined by the electrode–cell interface. Consequently, standard impedance platforms are less suited for immune-competent OoC systems that incorporate physiologically relevant 3D architectures or weakly adherent target populations. To extend impedance sensing into such contexts, flexible and conformable electrode designs have been developed to interface more effectively with organoid geometries, where more spatially distributed and minimally invasive monitoring of physiological changes in 3D tissue constructs is achieved [94,95]. When integrated with microfluidic platforms, these approaches offer improved compatibility between kinetic cytotoxicity readouts and 3D, immune-competent OoC architectures used for immunotherapy evaluation.

### 5.4. Summary

Emerging sensing modalities extend OoC capabilities by enabling continuous monitoring of immune activation kinetics, microenvironmental states, and functional cytotoxicity. However, their integration introduces additional engineering constraints, including sensor stability, spatial placement, and compatibility with flow and tissue organization. These tradeoffs reinforce a central principle: sensing strategies must be co-designed with platform architecture, transport conditions, and biological objectives rather than appended as independent modules. Consequently, effective OoC systems require coordinated integration of immune components, measurement workflows, and microenvironmental control, which forms the basis for the system-level design considerations discussed in Section 6.

## 6. Translational Considerations and Future Engineering Directions

Animal models have been foundational in immunotherapy research, but interspecies genetic and physiological differences limit predictive accuracy for certain immuno-oncology outcomes [96,97]. OoC systems provide a complementary strategy by enabling human-relevant tissue interactions and immune dynamics under controlled transport and microenvironmental conditions. Early successes have demonstrated that these systems can recapitulate system-level drug responses in vitro [98]. Building on these advances, future progress will depend on engineering rigor, quantitative validation, and scalable system design.

### 6.1. Standardization, Reproducibility, and Fit-for-Purpose Validation

The limited reproducibility of current OoC platforms remains a primary barrier for rigorous evaluation and cross-study comparability. Variability in fabrication methods, cell sourcing, and operating conditions introduces inconsistencies that complicate interpretation of immune responses and reduce confidence in experimental outcomes [99]. Standardization of key operating parameters, including shear stress, nutrient delivery, oxygenation, and immune cell dosing, is therefore a near-term priority to enable consistent system behavior for meaningful comparisons across studies. At the same time, validation should follow a fit-for-purpose framework in which platform performance is evaluated relative to a defined application. This requires benchmarking OoC outputs against well-annotated clinical and/or reference datasets to establish relevance for specific immunotherapy questions [99]. Incorporating donor-derived primary and iPSC-based cells further improves generalizability by capturing inter-individual variability, particularly in immune-mediated endpoints such as cytokine release and cytotoxic responses. Therefore, standardized operation and context-specific validation must be established to support reliable and interpretable OoC studies.

### 6.2. Integrated Design of Immune-Competent OoC Systems: Co-Design Across Biological Scales

#### 6.2.1. Biology-Driven Design: Tumor Heterogeneity and Immune Dynamics

The biological complexity of cancer immunotherapy is defined by a multi-dimensional landscape of spatial heterogeneity and temporal dynamics. Spatially, the tumor microenvironment presents varying degrees of immune infiltration (immune-inflamed versus immune-desert), stromal density, vascular accessibility, and hypoxia-driven immunosuppression, all of which determine therapeutic accessibility. These spatial factors are intrinsically connected to the distinct requirements of specific immune cells: T cells often require appropriate antigen presentation and cytokine support, while NK cells and macrophages are sensitive to local immunosuppressive signaling and microenvironmental cues [100,101,102]. Since such complexity cannot be adequately captured by homogeneous or well-mixed systems, OoC architectures must be engineered to preserve the spatial compartmentalization and transport gradients required for authentic cell–cell interactions.

Temporally, immune systems respond over distinct temporal regimes that impose contrasting measurement requirements. Acute activation (minutes or hours) is characterized by rapid cytokine release, early activation markers, and short-term cytotoxicity, which necessitates high-resolution kinetic assays. In contrast, chronic or sustained activation (weeks or months) involves phenotype evolution, functional exhaustion, and long-term persistence that requires platforms capable of stable operation and continuous, non-invasive monitoring. Together, these spatial and temporal distinctions establish the fundamental functional constraints for both platform architecture and assay selection, ensuring that engineered systems remain relevant to human clinical outcomes.

#### 6.2.2. Mechanisms-Driven Design: Mapping Immunotherapies to OoC Requirements

The design of immune-competent OoC platforms must be guided by the dominant mechanism of the therapy under investigation, as different treatment classes impose distinct engineering constraints. Adoptive cell therapies, such as CAR-T and NK-based approaches, rely on a sequential cascade of physical processes: immune cell trafficking, endothelial adhesion, transmigration, and infiltration into 3D tumor matrices. These processes require vascularized and compartmentalized architectures that can support controlled flow and barrier function, while providing the spatial depth necessary to resolve 3D migration kinetics. In contrast, immune checkpoint blockade primarily modulates T cell functional state rather than its initial recruitment. The engineering priority shifts from structural complexity to temporal resolution. Platforms should be capable of monitoring cytokine dynamics, activation states, and exhaustion phenotypes over extended durations. Bispecific antibodies introduce a third set of requirements centered on spatial proximity and stoichiometry. Because their efficacy depends on bridging effectors and targets, these therapies are best served by architectures with tunable spatial organization that allow for precise control over effector-to-target ratios and cell–cell contact frequency.

These distinctions suggest that OoC platform design should be guided not only by general physiological realism, but also by the dominant mechanism of the immunotherapy under investigation. Accordingly, architecture and assay selection must be aligned with the specific biological processes that govern therapeutic efficacy.

#### 6.2.3. Architecture–Assay Co-Design: Constraints and Tradeoffs

A central theme emerging across immune-competent organ-on-a-chip (OoC) systems is that platform architecture, immune cell incorporation, and measurement strategies cannot be designed independently. Instead, they form a tightly integral system in which each component constrains and enables the others. Consequently, OoC development is best framed as a problem of architecture–assay co-design, where biological objectives, transport conditions, and measurement workflows are jointly optimized to preserve immune function while maintaining physiologically relevant microenvironments. This systems-level perspective requires balancing the physical requirements of the tissue, such as spatial organization, nutrient delivery, and operational conditions, with the technical requirements of the intended readouts.

From the perspective of immune cell incorporation (Section 3), the choice of delivery mode and spatial organization directly defines the structure and dynamics of immune engagement. Vascular perfusion models enable physiologically relevant trafficking and extravasation, yet they require carefully tuned flow conditions to balance nutrient delivery against shear-induced cell activation. These perfused systems facilitate longitudinal sampling of secreted factors but may dilute transient signals. In contrast, compartmentalized architectures regulate immune exposure and preserve the localized signaling gradients necessary to study polarization and exhaustion without premature cell depletion. However, these low-flow or even static configurations often complicate repeated sampling and system-level quantification. Thus, immune incorporation strategies chosen to preserve biological fidelity simultaneously define the spatial and temporal limits of the data that can be collected.

These architectural constraints directly influence the implementation of immunological assays (Section 4) and emerging sensors (Section 5). Classic assays like fluorescence imaging and flow cytometry provide high-dimensional insights but exhibit significant tradeoffs in microfluidic environments: imaging is limited by optical access, phototoxicity, and analysis complexity in 3D tissues, while flow cytometry often requires destructive sample preparation, therefore constraining it to endpoint analysis. Integrated sensors provide a path toward continuous, non-invasive monitoring of kinetic state variables, like oxygen and cytokines, yet they introduce new engineering tensions. For instance, in situ sensing improves local fidelity at the cost of fabrication complexity and potential perturbation of tissue organization and flow fields. Conversely, effluent-based measurements are minimally disruptive but report spatially averaged signals that may mask critical local heterogeneities.

These findings conclude that no single modality is sufficient to capture the full spectrum of immune behavior in OoC systems. Instead, effective platforms rely on complementary combinations of assays, selected based on the underlying biological hypothesis, expected time scales, spatial complexity, and operational conditions. These findings also suggest that assay compatibility, including channel geometry, material selection, and sampling interfaces, should be considered during early-stage device design rather than as a later, sequential addition. By aligning architecture, immune function, and measurement strategy, OoC platforms can transition from descriptive mechanistic models toward robust, fit-for-purpose tools capable of generating the reproducible and interpretable data required for clinical immunotherapy development.

### 6.3. Translation and Adoption

Building on standardized operation and validated performance discussed in Section 6.1, translation of OoC systems into immunotherapy development pipelines depends on their demonstration within defined applications. From a regulatory perspective, OoC systems are increasingly positioned within the broader framework of New Approach Methodologies (NAMs) as complementary tools for preclinical evaluation, particularly in mechanistic studies and safety assessment. Meanwhile, OoC platforms must demonstrate that their outputs provide actionable insights relative to existing models. This includes alignment with clinically relevant endpoints and the ability to assist decision-making in areas such as immunotoxicity or therapeutic response. Transparent reporting of device design, operating conditions, and assay workflows is necessary to support interpretation and cross-study comparison.

Although formal regulatory pathways remain under development, current applications suggest that well-characterized OoC systems can contribute to preclinical evaluation when their performance and limitations are clearly defined. Adoption is therefore likely to be application-specific, with the greatest impact in areas where OoC platforms provide unique physiological or mechanistic insight.

### 6.4. Scalability, Manufacturability, and Operation

Translation to routine use requires the technical transition from laboratory-scale prototypes to robust and manufacturable systems. While early systems relied heavily on PDMS for rapid prototyping, translation toward industrial use increasingly favors thermoplastic materials and glass compatible with injection molding and high-throughput fabrication [103,104]. Additionally, modular and cartridge-based designs have been reported to further improve scalability and facilitate integration into automated workflows [49,105].

In operating OoC systems, stable flow control (e.g., pressure-driven or peristaltic pumping) is critical for maintaining consistent shear stress and residence time distributions while mitigating bubble formation and channel fouling. Long-term operation further requires careful management of evaporation, media replenishment, and device sealing to ensure reproducibility over multi-day or multi-week experiments.

### 6.5. Data Integration and Computational Analysis

The increasing complexity of OoC datasets, spanning time-resolved imaging, multiplexed cytokine measurements, and multi-parameter sensor outputs, has motivated the adoption of data-driven approaches such as machine learning and deep learning [106,107]. These methods are particularly effective for extracting quantitative features from high-content imaging data, including immune cell tracking, infiltration dynamics, and cell–cell interaction patterns that are difficult to analyze manually. Machine learning can also be applied to integrate multi-modal datasets (e.g., cytokine profiles, oxygen dynamics, and cytotoxicity readouts) to identify predictive signatures of therapeutic responses or resistance. When combined with mechanistic models, the resulting hybrid approaches provide the potential for interpretable and physiologically grounded predictions. Nevertheless, broader adoption of artificial intelligence in OoC remains limited by the availability of standardized datasets, challenges in model interpretability, and the lack of unified data formats across platforms.

## 7. Conclusions

In summary, engineering organ-on-a-chip systems for cancer immunotherapy requires co-optimization of (i) platform architecture and operating mode, (ii) immune cell incorporation strategies that preserve function and spatial biology, and (iii) assay workflows that provide interpretable, fit-for-purpose readouts. Continued progress in standardization, validation, and scalable fabrication will determine how rapidly immune-competent OoC platforms transition from specialized demonstrations to widely adopted tools in immunotherapy research and development.

## Figures and Tables

**Table 1:** Major milestones in the development of OoC for immunotherapy research.

Period	Stage/Milestone	Key Achievements	Immunotherapy Relevance	Ref.
2000–2010	Microfluidic control of transport	Flow control; gradient generation; shear modeling; PDMS rapid prototyping	Immune cell migration/chemotaxis under defined gradients; early dynamic co-culture concepts	[31,32]
2010–2014	Organ-level barrier models under perfusion/mechanics	Barrier-forming epithelial/endothelial interfaces; mechanical actuation; sustained perfusion	Leukocyte–barrier interactions; cytokine-driven inflammation in tissue-relevant geometries (early immune–tissue coupling)	[20,34,45]
2015–2018	Immune integration + compartmentalized co-culture	Immune–endothelial/tissue co-culture; ECM/hydrogel compartments; controlled immune delivery and trafficking paths	Tumor–immune crosstalk models; immune infiltration/extravasation assays; early evaluation of immunomodulators in human-relevant microenvironments	[46,47,48]
2018–2021	Recirculation and multi-compartment/multi-organ coupling	Recirculating perfusion; automated pumping/valving; cross-chip sampling; multi-week operation	System-level immune crosstalk; off-target immune effects; distributed exposure scenarios relevant to PK/PD hypotheses (still mechanistic, not regulatory)	[49,50,51]
2020–present	Translation-oriented platform engineering	Improved robustness/repeatability; standardized workflows; modular immune “add-ons”; increased use of patient-derived cells/organoids	More reproducible immunotoxicity and cytokine-related workflows; better comparability across studies; fit-for-purpose screening and mechanism testing	[8,44]

**Table 2:** Representative immunological readout assays for OoC systems.

Assay Type	Measurement	Temporal Resolution	OoC Compatibility	Invasiveness	Representative Immunotherapies	Relevant References
Live-cell imaging (e.g., fluorescence, confocal)	Immune trafficking, tumor infiltration, cell–cell interactions	High (continuous)	High	Non-invasive	CAR-T, Bispecific antibodies, Checkpoint Inhibitors	[41,63,64,65]
Effluent ELISA/Multiplex	Secreted cytokines (e.g., IFN-γ, IL-2, TNF-α, leptin)	Periodic (discrete)	High	Minimally invasive	Checkpoint Inhibitors, Cytokine Therapies	[35,41,67]
Flow cytometry	Immunophenotype, activation, exhaustion markers	Endpoint	Low	Invasive	CAR-T, Checkpoint Inhibitors, Cancer Vaccine	[70,71]
Electrochemical sensing	Cytokine levels, immune checkpoints	High (continuous)	High	Non-invasive	CAR-T, NK, Checkpoint Inhibitors, Bispecific Antibodies	[72,73,74]
Metabolic sensors	Local microenvironment (e.g., O_2_, pH)	High (continuous)	High	Non-invasive	CAR-T, Checkpoint Inhibitors	[75,76,77,78]
Electrical impedance sensing (e.g., TEER)	Cell barrier integrity, cell viability, morphology, and adhesion	High (continuous)	High	Non-invasive	CAR-T, Oncolytic Virus Therapy	[79,80,81,82]

## Data Availability

No new data were created or analyzed in this study.
